# Parthenocarpy in *Cucurbitaceae*: Advances for Economic and Environmental Sustainability

**DOI:** 10.3390/plants12193462

**Published:** 2023-10-02

**Authors:** Shouwei Tian, Zeliang Zhang, Genji Qin, Yong Xu

**Affiliations:** 1 State Key Laboratory of Vegetable Biobreeding, Beijing Vegetable Research Center, Beijing Academy of Agriculture and Forestry Science, Beijing 100097, China; 2 State Key Laboratory of Protein and Plant Gene Research, School of Life Sciences, Peking University, Beijing 100871, China

**Keywords:** *Cucurbitaceae*, parthenocarpy, phytohormone, genetic regulation, molecular breeding

## Abstract

Parthenocarpy is an important agricultural trait that not only produces seedless fruits, but also increases the rate of the fruit set under adverse environmental conditions. The study of parthenocarpy in *Cucurbitaceae* crops has considerable implications for cultivar improvement. This article provides a comprehensive review of relevant studies on the parthenocarpic traits of several major *Cucurbitaceae* crops and offers a perspective on future developments and research directions.

## 1. Introduction

The *Cucurbitaceae* family encompasses numerous commercially important crops that yield fruits known as cucurbits, including cucumber (*Cucumis sativus* L.), melon (*Cucumis melo* L.), watermelon (*Citrullus lanatu*s L.), squash/pumpkin (*Cucurbita* spp.), bitter gourd *(Momordica charantia* L.), and bottle gourd (*Lagenaria siceraria* L.) [1]. As with other monoecious plants, successful fruiting in cucurbits depends on favorable pollination conditions. Yield may be reduced in the absence of pollinators or under unfavorable environmental conditions, such as a lack of light, a high humidity, or an elevated temperature. Parthenocarpy could potentially overcome the problem of poor fruit set resulting from unfavorable pollination conditions [2].

Parthenocarpy induces fruit set and development without the need for pollination and fertilization [3], thereby enhancing the fruit set and ensuring consistent fruit production in adverse conditions. Parthenocarpic fruits are seedless, which is popular among consumers, because seedlessness improves flavor and enables a longer shelf life, and consumers do not need to remove seeds [4]. In addition, parthenocarpy greatly reduces the labor of artificial pollination frequently required to increase the yields of cross-pollinated *Cucurbitaceae* crops. Therefore, parthenocarpy is a highly beneficial agronomic trait for crops within the *Cucurbitaceae* family. 

Parthenocarpy is classified as either natural or induced, depending on whether external stimuli are required. Natural parthenocarpy can be divided into two forms: obligate and facultative. Obligately parthenocarpic plants, such as pineapple (*Ananas comosus*), always produce seedless fruits, meaning that they must propagate through vegetative organs; in facultative parthenocarpy, seedless fruits only develop if pollination and fertilization are prevented (e.g., cucumber) [5]. Facultative parthenocarpy has greater potential for the breeding of *Cucurbitaceae* crops, which are usually propagated through seeds [6].

This review provides an overview of the current knowledge on the genetic mechanisms and germplasm resources available for *Cucurbitaceae* crops. Parthenocarpy is a complex trait governed by multiple genes and hormone signaling pathways that regulate fruit development. A comprehensive understanding of the genetic control of parthenocarpy is therefore vital for breeding programs to improve *Cucurbitaceae* cultivars with reliable and high yields. Finally, we address technical gaps and propose future directions for research in the field of parthenocarpic fruit formation in cucurbits.

## 2. Germplasm and Genetic Mechanism of Parthenocarpy in *Cucurbitaceae* Crops

*Cucurbitaceae* is a large family of plants with more than 130 genera and 800 species known all around the world. The identification and characterization of parthenocarpic cultivars could benefit the research and breeding of the parthenocarpic trait in *Cucurbitaceae* crops.

### 2.1. Cucumber (Cucumis sativus L.)

Cucumber is cultivated as a vegetable worldwide. As the first crop plant recorded to exhibit parthenocarpy [3], cucumber has become a model organism for studying parthenocarpy, owing to its abundant germplasm. In an investigation of 201 cucumber germplasms representing various ecotypes, 124 accessions had a parthenocarpy rate of 50% or more and only 12 lines were identified as non-parthenocarpic. European greenhouse cucumbers exhibited a generally higher rate of parthenocarpy, whereas XSBN-type cucumbers showed the least parthenocarpy [2].

The genetic mechanism regulating the parthenocarpy in cucumbers is still unclear. According to Pike and Peterson [7], this trait may be controlled by an incompletely dominant gene, *P*. The expression of the *P* gene in cucumber determines the occurrence of parthenocarpy, with plants that are homozygous dominant (*PP*) or heterozygous (*Pp*) expressing this gene and producing seedless fruits. Studies of two commercially cultivated parthenocarpic genotypes, PPC-6 and PPC-2, determined that a single incompletely dominant gene controls the trait of parthenocarpy, despite the significant influence of genetic background [8,9]. However, additional studies have indicated that the process of parthenocarpy in cucumber is quantitatively inherited and regulated by multiple genes [10,11,12,13,14].

### 2.2. Cucurbita Species

*Cucurbita* species are important fruit vegetables that supply carbohydrates, minerals, and vitamins. There are five domesticated species: *Cucurbita argyrosperma*, *Cucurbita ficifolia*, *Cucurbita maxima*, *Cucurbita moschata*, and *Cucurbita pepo* [15]. The identification and characterization of parthenocarpic cultivars could benefit the research and breeding of the parthenocarpic trait in *Cucurbita* species.

‘Whitaker’ is a summer squash (*C. pepo* L.) cultivar created by researchers from Cornell University. This cultivar is resistant to three viral diseases and sets parthenocarpic fruit, but the origin of its parthenocarpy is uncertain [16]. Research has indicated that its parthenocarpy is controlled by a single locus with incomplete dominance [17]. An evaluation of the parthenocarpic tendency of 48 long-fruited accessions of *C. pepo* subsp. *pepo* identified ‘CpCAL112’, ‘CM-37’, ‘E-272032’, ‘PI261610’, and ‘V-185’ as exhibiting the fastest parthenocarpic fruit growth [18]. ‘Miyazakiwase No. 1’ is the first parthenocarpic cultivar reported in *C. moschata*. Parthenocarpic fruit development does not have an adverse effect on fruit expansion or quality. Additionally, low temperatures in fall may increase the ability of ‘Miyazakiwase No. 1’ to set parthenocarpic fruits [15].

### 2.3. Melon (Cucumis melo L.)

Parthenocarpic melon resources are currently limited, and there are no commercial varieties available. A screen of 172 accessions from an east Asian melon collection identified 14 accessions with a strong parthenocarpic ability. Crosses between parthenocarpic accessions and a non-parthenocarpic cultivar, as well as among parthenocarpic accessions, suggest that parthenocarpy may be inherited in a recessive manner and is likely controlled by the same gene or genes in these accessions [19]. The shape, color, and sugar contents of the fruit from these parthenocarpic accessions differ greatly from those of commercial cultivars, indicating that much effort will be needed for breeding new cultivars with both a parthenocarpic ability and high fruit quality. 

Although parthenocarpy has been studied in *Cucurbitaceae* for many years, few germplasm resources are available beyond cucumber. For example, no parthenocarpic material has been reported in watermelon. The identification and development of abundant parthenocarpy resources will provide a cornerstone for studying the genetic and molecular mechanisms of parthenocarpy, as well as for the selection and breeding of novel parthenocarpic cultivars.

## 3. Artificially Induced Parthenocarpy in *Cucurbitaceae*

The induction of parthenocarpy is a common agricultural practice for producing seedless fruits and reducing the cost of manual pollination in cucurbit crops.

### 3.1. Induction of Parthenocarpic Fruit by Hormone Application

Auxin was the first hormone reported to induce parthenocarpy in plants. Auxin analogues induce parthenocarpy in *Cucurbitaceae* crops such as watermelon, cucumber, and zucchini (*C. pepo* L.), resulting in seedless fruit formation [20,21]. When applying auxin analogues externally to induce parthenocarpy in watermelon, the method of this application should be considered. The local application of auxin analogues to the watermelon ovary results in seedless watermelons with undesirable phenotypes, such as a smaller fruit size, thicker rind, and hollow cavities. However, when spraying over the entire plant, the resulting seedless watermelons show no significant difference from watermelons produced by pollination [22]. Qian et al. [23] successfully induced parthenocarpy in cucumbers by applying the exogenous plant growth regulator naphthaleneacetic acid (NAA) 1 day before and on the day of flowering.

Gibberellin (GA) is another crucial plant hormone acting downstream of auxins in the control of plant parthenocarpy [24,25]. Different gibberellins vary in their capability to induce parthenocarpy across species. For instance, GA3 strongly induces seedless fruit formation in tomatoes (*Solanum lycopersicum*) [26], but does not effectively promote parthenocarpy in cucumbers [23]. Conversely, parthenocarpic cucumbers can be obtained through treatment with gibberellin GA4+7. Cucumbers treated with GA4+7 show no significant weight difference compared to pollinated cucumbers. However, after storage, GA4+7-treated cucumbers have less firm flesh and higher concentrations of total flavonoids and proteins compared to pollinated cucumbers [23].

Cytokinin (CK) is another pivotal plant hormone promoting parthenocarpy in plants. Spraying *N*-(2-chloro-4-pyridyl)-*N*′-phenylurea (CPPU), an exogenous CK growth regulator, stimulates cell division. Hayata et al. [27] first employed CPPU to treat unpollinated watermelon ovaries, resulting in seedless fruit formation. Moreover, a concentration of 200 g/m^3^ CPPU did not impact the shape, soluble solids content, or rind thickness of the watermelon fruit, suggesting its direct applicability in production [22,27]. Watermelons treated with CPPU may exhibit a slight reduction in their sugar content compared to pollinated watermelons; however, this could be attributable to varietal differences [28,29]. CPPU is widely employed in the stimulation or augmentation of parthenocarpy in the cultivation of Cucurbitaceae crops, including cucumber [23], melon [30,31], pumpkin [32], and watermelon [22]. This application effectively enhances fruit setting, facilitates fruit enlargement, and consequently amplifies overall yield.

Other plant hormones can also induce parthenocarpy in *Cucurbitaceae* crops. The inhibition of ethylene biosynthesis or response induces the parthenocarpic development of fruit in zucchini squash [33,34]. The application of exogenous brassinosteroids (BRs) (24-epibrassinolide, EBR) induces parthenocarpic growth accompanied by active cell division in cucumber cultivars without a parthenocarpic capacity, whereas treatment with a BR biosynthesis inhibitor (brassinazole, BRZ) inhibits the fruit set in cultivars with a natural parthenocarpic capacity [35].

### 3.2. Pollination-Induced Parthenocarpy

Pollination with inactivated or incompatible pollen stimulates fruit enlargement, resulting in seedless fruits. These fruits do not show significant differences in quality or yield compared to fruits derived from regular pollination. The application of diploid watermelon pollen to the stigma of triploid watermelon induces fruit development and produces seedless watermelons [29]. Pollinating female flowers with pollen grains that have been irradiated with X-rays or gamma rays leads to parthenocarpic seedless diploid watermelons [36,37]. Sugiyama et al. [38] observed that pollinating watermelons with bottle gourd pollen can induce parthenocarpy, yielding seedless watermelons, whereas pollen from other *Cucurbitaceae* crops does not trigger parthenocarpy in watermelons. However, bottle gourd produces fruit by parthenocarpy after pollination not only with watermelon pollen, but also with pollen from various cucurbit species. The induction of parthenocarpy via stimulation with pollen from a distantly related genus has not been reported in other plant families and seems to be unique to *Cucurbitaceae*. The study of the parthenocarpic phenomenon during intergeneric pollination will not only be useful for investigating the mechanism of parthenocarpy, but will also provide useful information for research into the evolutionary history of *Cucurbitaceae* and for breeding seedless varieties [39].

### 3.3. Environmental Induction of Parthenocarpy in Cucurbit Crops

Environmental factors such as temperature, photoperiod, light intensity, and nutritional conditions have a significant effect on parthenocarpy [2]. Low temperatures can induce parthenocarpy in *Cucurbitaceae* crops such as zucchini, melon, and cucumber [40,41,42]. The auxin content in cucumber ovaries increases under nightly low temperatures, inducing cucumber parthenocarpy. Conversely, high temperatures inhibit the onset of parthenocarpy by suppressing auxin and gibberellin biosynthesis in cucumber ovaries [43]. Short-day conditions also promote parthenocarpy in cucumber by increasing auxin activity [44]. An adequate nutrient supply is a prerequisite for fruit development and plays a crucial role in parthenocarpy [2].

## 4. Genetic Regulation of Parthenocarpy

The exploration and functional validation of the genes related to parthenocarpy in *Cucurbitaceae* crops have not been extensively reported. Table 1 summarizes and organizes the currently known genes regulating plant parthenocarpy, to serve as a reference for further research on parthenocarpy in *Cucurbitaceae* crops.

### 4.1. Auxin-Related Genes

After pollination and fertilization, the level of indole-3-acetic acid (IAA; the primary active auxin) in the ovary immediately increases, activating the auxin signaling pathway essential for fruit set [45]. Parthenocarpy can result from alterations in the genes involved in auxin biosynthesis, polar transport, or signaling pathways. For example, the tryptophan monooxygenase, iaaM, can convert tryptophan into indole-3-acetamide (IAM), which is subsequently converted into IAA. The ovule-specific expression of the *iaaM* gene using the promoter of the *DefH9* gene from *Antirrhinum majus* enhances auxin biosynthesis, resulting in natural parthenocarpy in plants such as tobacco (*Nicotiana tabacum*), eggplant (*Solanum melongena*), cucumber, and strawberry (*Fragaria* × *ananassa*) [46,47,48].

**Table 1:** Genes involved in regulating parthenocarpic fruit formation.

	Gene Name	Genetic Modification	Phenotypic Manifestation	References
Auxin-related genes	*DeH9-iaaM*	Ovule-specific transgenic expression	Early fruit growth, normal size and shape	[46,47,48]
	*Pad-1*	RNAi	Normal fruits	[49]
	*SlPIN4*	RNAi	Sterile or facultative parthenocarpy	[50]
	*SlTIR1*	Overexpression	Severely dwarfed and exhibiting a fertility defect	[51]
	*SlIAA9*	RNAi	Altered leaf morphology and multiple organ fusion	[52]
	*SlARF5*	RNAi	Smaller size and lower weight	[53]
	*SlARF2A*	RNAi	Delayed fruit ripening	[54]
	*SlARF7*	RNAi	Curled leaves; stems with random growth orientation	[55]
	*AtARF8*;*SlARF8*	Loss of function	Significant increase in number and size of parthenocarpic fruit	[56,57]
	*SlAucsia*	RNAi	Leaf fusions and reflexed leaves	[58]
Gibberellin-related genes	*GA20ox1*	Overexpression	Longer hypocotyls and roots; taller plants with longer internodes and thinner stems	[59]
	*GA2ox*	RNAi	Significant inhibition of branching	[60]
	*SlDELLA*	Amino acid change or RNAi	Longer hypocotyls and shorter roots; slender plants with longer internodes and thinner stems	[61,62,63]
	*FveRGA1*	Loss of function	Larger and taller receptacle at stages 4 and 5	[64]
Other hormone-associated genes	*SlETR1*	Ethyl methane sulfonate, EMS	Smaller and elongated fruits	[65]
	*SlTPR1*	Overexpression	Altered fruit morphology	[66]
	*SlNCED1*	Overexpression	Serious malformation; base of style appears to have no definite shape; pistils split with multiple fruits forming from a single ovary	[67]
	*SlTPL1*	RNAi	Normal fruits; increased fruit set rate under heat stress	[68]
FIS-PRC2 complex	*AtFIE*	EMS	Female gametophytic lethal; 50% seed abortion in heterozygous plants	[69,70]
	*AtMEA*	EMS	Homozygous progeny is embryo-lethal	[71]
	*AtFIS2*	EMS	Initiation of seed development in absence of fertilization	[70]
	*AtMSI*	EMS	Initiation of seed development in absence of fertilization	[72]
Floral- or reproductive-development-associated genes	*MdPI*	Retrotransposon insert	Flowers have no petals or stamens but increased sepals and styles; normal fruit size	[73]
	*SlSEP1/TM29*	RNAi	Sepallata-like flowers; ectopic shoots	[74]
	*SlSPL/* *HYDRA*	Small transposable insert	Complete male and female sterility; reduction of almost 40% in size and up to 80% in fruit weight	[75]
	*SlAGL6*	CRISPR/Cas9	Normal fruits; improved yielding under heat stress	[6,76]
	*SlTAP3*	EMS	Complete or partial conversion of stamens into carpelloid organs	[77]
Other genes	*SlHB15A*	EMS, CRISPR/Cas9	Smaller and more abundant fruits	[78]
	*miR159*	Overexpression	Shorter plants; normal fruits	[79]
	*SlHWS*	Amino acid change mutation	Reduction in fertility; flowering delay	[80]
	*SlSPFF*	Loss of function	Dwarfism of floral organs, male sterility, delayed flowering, altered axillary shoot development, and parthenocarpic production of small fruits	[81]
	*SlCIN7*	RNAi	Decreased pollen germination rate; normal fruits	[82]
	*CHS*	RNAi	Dull, smaller, and more reddish fruits	[83]

The *Pad-1* gene, encoding an amino transferase, negatively regulates auxin biosynthesis by catalyzing the conversion of indole-3-pyruvic acid (IPyA) into tryptophan (Trp). The inhibition of the *Pad-1* gene causes high levels of auxin to accumulate in the ovary, thus inducing parthenocarpy in eggplant and tomato plants [49]. The *PIN-FORMED 4* (*PIN4*) gene in tomato encodes an auxin polar transport protein and is specifically expressed in flowers and young fruits. The silencing of *PIN4* results in parthenocarpy in tomatoes [50]. TRANSPORT INHIBITOR RESPONSE 1 (TIR1), INDOLE ACETIC ACID (IAA), and AUXIN RESPONSE FACTOR (ARF) are the auxin receptor, transcriptional repressor, and core transcription factor, respectively, within the auxin signaling pathway [84]. The overexpression of *TIR1* in tomato induces a parthenocarpic phenotype [51]. A functional deficiency or loss of the tomato *IAA9* gene also results in parthenocarpy [52]. Mutations in the *ARF8* gene in *Arabidopsis* and tomato [56,57,85], as well as the knockout or silencing of *ARF2A* [54], *ARF5* [53], or *ARF7* [55] in tomato, lead to parthenocarpy in these plants. The *AUCSIA* (*auxin cum silencing action*) gene encodes a small peptide of 53 amino acids that suppresses auxin responses. When the *AUCSIA* gene is silenced in tomato, plants exhibit parthenocarpic fruit development, along with other auxin-associated phenotypes such as leaf fusions [58]. These studies highlight the central role of auxin in controlling fruit set. Parthenocarpy can be achieved by manipulating auxin functions at multiple levels, including its biosynthesis, signaling cascades, and transport.

### 4.2. Gibberellin-Related Genes

Following successful pollination and fertilization, the active GA levels increase in the ovary. This is associated with an up-regulation in the expression of GA biosynthetic enzymes such as GA 20-oxidase and a down-regulation in the expression of GA catabolic enzymes such as GA 2-oxidase [86]. Modifications in GA biosynthesis and signaling pathways also lead to parthenocarpy in plants. For instance, an overexpression of the *GIBBERELLIN 20-OXIDASE 1* (*GA20OX1*) gene, which encodes a key enzyme in GA biosynthesis, results in parthenocarpic fruits in both *Arabidopsis* and tomato [59]. Silencing the expression of the genes encoding GA catabolic enzymes, *GA 2-oxidases* (*GA2oxs*), leads to parthenocarpic fruit development in tomato [60]. Parthenocarpy in the natural parthenocarpic tomato mutants *pat-2*, *pat-3*, and *pat-4* results from the accumulation of high concentrations of gibberellins in the unpollinated ovary [87,88]. The natural parthenocarpic phenotype in the tomato mutant ‘*pro*’ is attributed to a point mutation in the GA receptor, *SlDELLA* [61,62]. In both *Arabidopsis* and tomato, the knockout or silencing of the core transcriptional repressor gene *DELLA*, pivotal in the GA signaling pathway, leads to parthenocarpy [63,89]. In diploid wild strawberry, a mutation of the *FveRGA1* gene, which encodes the DELLA protein, results in a parthenocarpic phenotype [64].

### 4.3. Other Hormone-Associated Genes

Research by Shinozaki et al. [65] found a correlation between tomato parthenocarpy and ethylene levels. Ethylene biosynthesis is elevated in unpollinated wild-type pistils, whereas the ethylene levels in pollinated pistils decrease progressively after flowering as the ovary diameter increases. A mutation in the ethylene receptor gene *ERT1* (*sletr 1-1* mutant) results in the development of parthenocarpic fruits. The *SlTPR1* gene encodes a tripeptide repeat protein that interacts with the ethylene receptor. An overexpression of *SlTPRl* in tomato results in constitutive ethylene responses and the acquisition of parthenocarpy [66]. Transcriptomic analyses have revealed that the genes involved in abscisic acid (ABA) biosynthesis and response are highly expressed in unpollinated mature tomato ovaries, with their expression decreasing after fruit set, suggesting a role for ABA in the transition from flower to fruit [90]. The *SlNCED1* gene, encoding 9-cis-epoxycarotenoid dioxygenase, is the primary ABA biosynthetic enzyme in tomato ovary. Ovary growth in *SlNCED1*-overexpressing lines starts before flowering, and 90% of the fruits from these lines are parthenocarpic [67]. Transcriptional co-repressors (TPL/TPRs) act as a central regulatory hub controlling phytohormone pathways. The down-regulation of *SlTPL1* results in facultative parthenocarpy upon emasculation and under heat stress conditions [68].

### 4.4. Genes Associated with the FIS-PRC2 Complex

Forward genetic screens under conditions that prevent pollen germination have identified mutants exhibiting parthenocarpy or fruit enlargement in the absence of pollination in *Arabidopsis*. These mutants include *fertilization-independent endosperm* (*fie*) [69], *fertilization-independent seed 1* (*fis1*), *fis2*, and *fis3* [70]. Further studies have revealed that *fis1* is an allelic mutant of the seed-sterile mutant *medea* (*mea*); the MEA protein, a core member of the Polycomb Repressive Complex 2 (PRC2), contains a characteristic SET domain that confers histone methyltransferase activity [71]. Subsequent gene cloning revealed that both FIE and FIS2 are members of the PRC2 complex. Mutations in these genes lead to similar phenotypes, characterized by parthenocarpy, the autonomous division and development of the central cell, forming spontaneous endosperm, and seed coat enlargement without pollination. Upon fertilization, these mutants also show seed sterility, with the embryo arrested at the heart stage and the endosperm uncellularized, while the silique elongates normally, leading to seed-sterile parthenocarpic fruit [69,70]. Reverse genetics studies on another member of the PRC2 complex, MULTICOPY SUPPRESSOR OF IRA 1 (MSI1), revealed that the *msi1* mutant has phenotypes similar to those of the *fis* mutants [72]. The PRC2 complex is responsible for fertilization-independent endosperm formation and parthenocarpy by repressing the high expression of the downstream MADS-box gene *PHERES1* (*PHE1*) in the central cell and post-fertilization endosperm nuclei, thereby regulating their division [91]. Furthermore, PHE1 directly binds to the promoter regions of several genes, including the gene encoding the key auxin biosynthetic enzyme YUCCA10 (YUC10), as well as another MADS-box gene, *AGAMOUS-LIKE62* (*AGL62*), to regulate seed and fruit development [92]. In *fis2* and *fie* mutants, the *YUC10* expression is increased in the central cell, leading to auxin accumulation, which results in spontaneous endosperm formation and parthenocarpy [93].

### 4.5. Genes Related to Floral or Reproductive Development

Fruits develop primarily from the fourth whorl organ, the ovary, or associated parts of the flower [94]. Scientists have therefore identified several mutations in the genes controlling floral or reproductive development that lead to parthenocarpy by screening and analyzing the plant mutants capable of natural parthenocarpy. For example, in apple (*Malus domestica*), a transposon insertion causes a mutation in the B-class gene *PISTILLATA* (*PI*) of the ABCDE model of floral development, resulting in parthenocarpic fruit formation [73]. In tomato, the down-regulation of the B-class MADS-box gene *APETALA 3* (*AP3*) results in male sterility and the development of parthenocarpic fruits [77]. Reducing the expression level of the E-class gene *SEPALLATA 1* (*SEP1*)/*TM29* also induces parthenocarpy [74]. An analysis of the tomato parthenocarpic mutant *hydra* revealed that a loss of function of *SPOROCYTELESS* (*SPL*)/*HYDRA*, which controls the fate of megaspore mother cells, confers parthenocarpic ability in tomato [75]. Similarly, an analysis of the tomato *sg1* mutant revealed that a mutation in the tomato MADS-box gene *SlAGL6* results in the formation of seedless parthenocarpic fruits [6]. The *slagl6* mutant forms seedless fruits with a normal appearance without relying on fertilization, while retaining its ability to reproduce sexually at high temperatures. *SlAGL6* accumulates predominantly in the integuments of ovules, and its absence leads to an over-proliferation of the integuments and the aberrant development of the innermost layer, the endothelium [76].

### 4.6. Other Genes

*SlHB15A* encodes a Class III homeodomain-leucine zipper (HD-ZipIII) protein that modulates hormone biosynthesis to prevent tomato fruit set prior to pollination. In the absence of fertilization, *SlHB15A* suppresses auxin biosynthesis and activates ethylene biosynthesis, thereby maintaining the ovary in a growth-arrested stage. The inactivation of *SlHB15A* or pollination reverses this process, resulting in the accumulation of auxin and the inhibition of the ethylene response. Missense or null mutations in *SlHB15A* cause parthenocarpy in tomato [78]. An overexpression of *SlMIR159* in tomato results in parthenocarpy and early fruit ripening. In plants overexpressing *SlMIR159*, the silencing of *SlGAMYB1/2* leads to a dysregulation of the pathways associated with ovule and female gametophyte development, as well as auxin signal transduction [79]. The tomato *slhws-1* mutant exhibits thermo-sensitive parthenocarpy, with a higher parthenocarpic rate under warmer conditions. The *slhws-1* mutant phenotype results from an amino acid mutation in the coding region of the *Solyc01g095370* gene, which encodes an F-box protein [80]. The tomato gene *Solyc04g077010* encodes a receptor-like protein kinase, and its loss-of-function mutation results in parthenocarpy, ovarian cell enlargement, and increased expression of the GA metabolism gene *GA20ox1* [81]. *SlCIN7* encodes a sucrose-cleaving invertase, and the silencing of the *SlCIN7* gene increases the reactive oxygen species levels in tomato pollen grains, reduces pollen viability, and leads to the formation of parthenocarpic fruits [82]. RNA interference (RNAi) targeting of the first gene in the flavonoid pathway, *chalcone synthase* (*CHS*), and the inhibition of the flavonoid biosynthetic pathway result in the production of parthenocarpic fruits [83].

## 5. Advances in Parthenocarpy Research Techniques in *Cucurbitaceae* Crops

Driven by rapid progress in the fields of molecular biology, genomics, and biotechnology, we have gained a deeper understanding of the molecular mechanisms and regulatory genes involved in parthenocarpy. The following section highlights the current technical advances in investigating parthenocarpy in Cucurbitaceae crops.

### 5.1. QTL Mapping and Identification of Parthenocarpy Candidate Genes

Cucumber is an essential model plant for studying parthenocarpy, owing to its abundance of parthenocarpic germplasm resources. The quantitative trait loci (QTLs) associated with the parthenocarpic traits in cucumber are currently identified using markers from linkage maps developed via sequencing, which allows for an accurate localization of these QTL loci on chromosomes. Wu et al. [12] utilized SSR and InDel markers to construct a linkage map for the QTL mapping of parthenocarpy in cucumber. They identified *Parth2.1* on chromosome 2 as the major-effect QTL for parthenocarpy and selected five and eight candidate genes for parthenocarpy using SSR and InDel markers, respectively. Lietzow et al. [13] identified seven parthenocarpic QTLs in the North American pickling cucumber line 2A. They discovered that parthenocarpy is regulated by one QTL each on chromosomes 5 and 7 (*parth5.*1 and *parth7.1*) and two QTLs on chromosome 6 (*parth6.1* and *parth6.*2). In the parthenocarpic PPC-6 genotype, a combination of QTL-seq analysis and conventional mapping with an F2:3 population revealed a major-effect QTL, *Parth6.1*, and two major-effect genes, *Csa_6G396640* and *Csa_6G405890*. These genes are related to auxin biosynthesis and response factors, respectively [8]. Gou et al. [2] evaluated the parthenocarpic ability of 201 cucumber germplasms through three independent experiments. Additionally, they pinpointed six genetic loci responsible for cucumber parthenocarpy using a genome-wide association study on re-sequenced genomic data from 31 cucumber lines.

### 5.2. Exploring Parthenocarpy-Related Genes Using RNA-Seq Technology

RNA sequencing (RNA-seq) is a high-throughput sequencing technology enabling the identification of transcriptomes and the quantification of gene expression. This technology facilitates the analysis of various processes occurring during a plant’s life cycle. Research on plant parthenocarpy using RNA-seq primarily involves identifying differentially expressed genes (DEGs), followed by studying the functions of these genes and associated genes in upstream and downstream regulatory networks.

Li et al. [95] conducted a transcriptomic analysis using RNA-seq on cucumber fruits 2 days post-pollination, naturally parthenocarpic fruits, artificially induced parthenocarpic fruits, and aborted fruits. They compared the DEGs between setting fruits and aborted fruits, and identified 14 genes predicted to be related to parthenocarpy. A transcriptional analysis of these candidate genes indicated cross-talk among auxin, cytokinin, and gibberellin during the parthenocarpic process. In a study of the fruit set and parthenocarpy in zucchini, Pomares-Viciana et al. [96] sequenced the reference transcriptome for the first time. A differential expression analysis allowed them to elucidate the coordinated role of the plant hormones in zucchini fruit set and parthenocarpy. Notably, auxin response factors, such as *ARF18*, transcriptionally activate the key GA biosynthesis gene *GA20ox1*, leading to a down-regulation of ethylene-biosynthesis-related genes such as *ACO1*, *ETR2*, *ERF11*, and *ERF17.* This study identified the role of auxin and GA interactions in suppressing ethylene biosynthesis during zucchini fruit set and parthenocarpy. A transcriptomic analysis of CPPU-induced melon parthenocarpy revealed that CPPU specifically induces GA-related genes during melon fruit set, with the gene encoding the key enzyme gibberellin 20-oxidase 1 (CmGA 20 ox 1) being upregulated. Further research has revealed that the gene encoding the two-component response regulator 2 (*CmRR2*) within the cytokinin signaling pathway is significantly expressed during fruit set and positively regulates the expression of *CmGA 20 ox 1*. This confirms that CPPU-induced melon fruit set depends on GA biosynthesis, providing a theoretical foundation for developing parthenocarpic melon germplasm [31].

### 5.3. Accelerating Parthenocarpic Breeding through Genetic Engineering Techniques

Parthenocarpy is a desirable trait for agricultural production, owing to its ability to decrease labor costs and produce the seedless fruits preferred by consumers. Nonetheless, natural parthenocarpic germplasm resources are limited to a few crops. For example, within the *Cucurbitaceae* family, only cucumbers have a rich parthenocarpic germplasm. Genetic engineering has shown that, by modifying a single gene, fruit growth can be decoupled from fertilization, leading to seedless fruits in horticultural plants. Several parthenocarpic tomato mutants have been obtained through techniques including overexpression, gene silencing, and gene editing [97]. A natural parthenocarpic germplasm was produced in cucumbers by inducing auxin biosynthesis through the expression of the *iaaM* gene in ovules, utilizing the promoter from the *DefH9* gene of *Antirrhinum majus* [46]. 

## 6. Future Directions

The development of strong parthenocarpy is an essential target trait in the breeding of *Cucurbitaceae* crops. However, the exploration and utilization of parthenocarpy genes have remained limited. Elucidating the molecular mechanisms underlying parthenocarpy, identifying the key regulatory genes, and enhancing the parthenocarpy trait in germplasm resources are fundamental approaches for breeding *Cucurbitaceae* crops. Several aspects related to parthenocarpy need to be urgently addressed in the future:

### 6.1. Exploration of Key Genes Regulating Strong Parthenocarpy in Cucumber

Research on the parthenocarpy in the model crop cucumber has been ongoing for years. However, the genes directly controlling the parthenocarpy in cucumber remain elusive. Although numerous studies have identified QTL intervals and potential candidate genes through mapping population constructions, there are significant discrepancies in the conclusions of different studies [2,8,9,12,13]. On the one hand, these differences might arise from the diverse germplasm materials used, suggesting that the genes controlling parthenocarpy might indeed differ in different germplasms. On the other hand, the environmental impact on this trait could be a contributing factor [2]. The further development of genetic markers, QTL mapping to predict parthenocarpy-related genes, and validation through transgenic experiments should be conducted under strictly controlled experimental conditions, such as day–night temperature and humidity. Gene editing has been established in cucumber, breaking through the limitations of specific cultivars [98,99]. The use of this technology to ascertain the regulatory roles of candidate genes in parthenocarpy and reveal genetic mechanisms will establish a theoretical basis for molecular breeding.

### 6.2. Development of Facultative Parthenocarpy Germplasm

More than 30 genes regulating plant parthenocarpy have been reported (as shown in Table 1). Many parthenocarpic materials exhibit exclusive parthenocarpy, where the plants are sterile or the parthenocarpic fruits are small and of poor quality, making them unsuitable for breeding and production. Only materials demonstrating facultative parthenocarpy with a satisfactory fruit size and quality have practical application value. For instance, the *Pad-1* gene in eggplant encodes an aminotransferase that catalyzes the conversion of indole-3-pyruvic acid (IPyA) into tryptophan (Trp), negatively regulating auxin biosynthesis. Suppressing the expression of *Pad-1* results in parthenocarpic fruits that exhibit no significant difference compared to wild-type materials pollinated normally [49]. In tomato, *Slagl6* mutants spontaneously produce seedless fruits that maintain a regular shape and weight [6]. In recent years, gene-editing technology for *Cucurbitaceae* crops has advanced significantly, with gene-editing techniques being established for cucumber [100], watermelon [101], melon [102], and pumpkin [98]. Leveraging gene-editing technology to create mutated versions of the aforementioned genes may facilitate the production of economically sustainable parthenocarpic materials within the *Cucurbitaceae* crop family.

### 6.3. Development of an Efficient Promoter-Editing System for Cucurbitaceae Crops

Parthenocarpy is the spontaneous initiation of fruit development in the absence of fertilization stimuli. Plant parthenocarpy can be promoted through the expression of genes that negatively regulate the initiation of fruit development, such as the *Pad-1* and *AGL6* genes mentioned above. Conversely, enhancing plant parthenocarpy can also be achieved by the tissue-specific overexpression of genes that initiate fruit development [46,48]. Commonly used overexpression techniques typically result in transgenic products, which face stringent regulations when introduced into the market. With the advancement of gene-editing technology, the up-regulation of gene expression without transgenes has become possible. Recently, Zhou et al. [103] developed a promoter-editing system named CAPE, based on CRISPR/Cas12a. This system combines a predictive model for critical promoter regions with a highly efficient CRISPR/Cas12a-based single/multi-gene-editing system. They successfully utilized the CAPE system in rice to target the *OsGBSS1* and *OsGS3* genes, creating a series of quantitative trait variation sets for grain starch content and grain size, respectively. Looking ahead, establishing such a promoter-editing system in *Cucurbitaceae* crops to linearly adjust the expressions of genes positively regulating parthenocarpy, without transgene dependency, will potentially pave the way for the development of commercially viable parthenocarpic germplasm materials.

## Data Availability

Not applicable to this article.
